# Supplementary material for: The genetic architecture of NAFLD among inbred strains of mice
Source: eLife. 2015 Jun 12;4:e05607. doi: 10.7554/eLife.05607 (PMC4493743; doi:10.7554/eLife.05607)
Supplement: Supplementary file 2. — Correlation of hepatic TG and plasma metabolites. DOI: http://dx.doi.org/10.7554/eLife.05607.025 [file elife05607s002.pdf]

Supplementary File 2. Correlation of hepatic TG and plasma metabolites.

Metabolite	bicor	p value
5-HIAA	-0.004	0.970
5'-adenosylhomocysteine	0.047	0.657
ADMA/SDMA	0.134	0.199
alanine	0.098	0.349
allantoin	0.105	0.315
alpha-glycerophosphocholine	-0.112	0.284
aminoisobutyric acid	0.056	0.594
anthranilic acid	0.020	0.851
arginine	-0.530	9.9E-12
asparagine	0.066	0.528
aspartate	0.059	0.574
betaine	-0.010	0.923
carnitine	0.022	0.831
choline	-0.028	0.791
cis/trans hydroxyproline	-0.018	0.863
citrulline	0.180	0.034
creatine	0.230	0.006
creatinine	0.250	0.003
dimethylglycine	0.061	0.560
GABA	-0.048	0.644
glutamine	-0.104	0.320
glycerol	0.126	0.229
glycine	0.183	0.078
histidine	0.093	0.376
isoleucine	-2.3E-04	0.998
kynurenic acid	0.047	0.656
leucine	0.017	0.869
lysine	0.032	0.759
methionine	0.075	0.472
N-carbomoyl-beta-alanine	0.044	0.678
niacinamide	-0.009	0.930
NMMA	0.046	0.661
ornithine	-0.180	0.027
phenylalanine	0.060	0.567
proline	0.199	0.055
serine	0.199	0.055
spermidine	0.016	0.877
taurine	0.089	0.399
thiamine	-0.244	0.018
threonine	0.157	0.132
thyroxine	-0.067	0.525
trimethylamine-N-oxide	0.180	0.034
tryptophan	-0.095	0.365
tyrosine	0.019	0.857
valine	0.078	0.455
